# Epoxy/Polycaprolactone Systems with Triple-Shape Memory Effect: Electrospun Nanoweb with and without Graphene *Versus* Co-Continuous Morphology

**DOI:** 10.3390/ma6104489

**Published:** 2013-10-09

**Authors:** Márta Fejős, Kolos Molnár, József Karger-Kocsis

**Affiliations:** 1 Department of Polymer Engineering, Faculty of Mechanical Engineering, Budapest University of Technology and Economics, Muegyetem rkp. 3., Budapest H-1111, Hungary; E-Mail: molnar@pt.bme.hu; 2 MTA-BME Research Group for Composite Science and Technology, Muegyetem rkp. 3., Budapest H-1111, Hungary; E-Mail: karger@pt.bme.hu

**Keywords:** epoxy, polycaprolactone, nanofiber, triple-shape memory effect, graphene, electrospinning, co-continuous phase structure, nanocomposite

## Abstract

Triple-shape memory epoxy (EP)/polycaprolactone (PCL) systems (PCL content: 23 wt %) with different structures (PCL nanoweb embedded in EP matrix and EP/PCL with co-continuous phase structure) were produced. To set the two temporary shapes, the glass transition temperature (*T_g_*) of the EP and the melting temperature (*T_m_*) of PCL served during the shape memory cycle. An attempt was made to reinforce the PCL nanoweb by graphene nanoplatelets prior to infiltrating the nanoweb with EP through vacuum assisted resin transfer molding. Morphology was analyzed by scanning electron microscopy and Raman spectrometry. Triple-shape memory characteristics were determined by dynamic mechanical analysis in tension mode. Graphene was supposed to act also as spacer between the nanofibers, improving the quality of impregnation with EP. The EP phase related shape memory properties were similar for all systems, while those belonging to PCL phase depended on the structure. Shape fixity of PCL was better without than with graphene reinforcement. The best shape memory performance was shown by the EP/PCL with co-continuous structure. Based on Raman spectrometry results, the characteristic dimension of the related co-continuous network was below 900 nm.

## 1. Introduction

Shape memory polymers (SMPs) change their shapes reversibly from temporary to permanent under combined action of mechanical load and external stimulus, which is in most cases heat. For thermosets, the transformation temperature is the glass transition temperature (*T_g_*). The temporary shape is set by deformation above *T_g_* followed by cooling under load. During this procedure, the segments between the crosslinks adapt to the external load via conformational rearrangements. The strain energy, stored by this way, is released when the material is unloaded and heated above its *T_g_* whereby the permanent shape is restored. The related SMPs are termed one-way, dual-shape memory systems because the reversible shape change occurs only from temporary to permanent. One-way SMP systems may, however, remember two or more temporary shapes before returning to the permanent one. In case of EP-based systems triple-shape memory functions has been triggered by two approaches so far, viz. (i) bilayer system of two EPs with different *T_g_* that are, however, well bonded to each other (“bimetal” principle) [1]; and (ii) EP containing an electrospun semicrystalline thermoplastic nanoweb [2]. The material of the latter was poly(ε-caprolactone) (PCL) the melting temperature (*T_m_*) of which was higher than the *T_g_* of the embedding EP matrix. Note that in this case *T_m_* of PCL served as a reversible switch for setting one shape, whereas the *T_g_* of the EP matrix served to set the other temporary shape in the memorizing cycle. A large body of works has already addressed different SMPs, and the related knowledge is well summarized in reviews [3,4]. However, the above-cited works were dealing with EP-based triple-shape memory systems. Considering the second approach, it is obvious that infiltration of the electrospun nanoweb is a challenging task, irrespective of whether this occurs under vacuum and/or pressure. To facilitate the infiltration process with the EP, the structural stiffness of the nanoweb should be enhanced. This may happen by incorporation of graphene in the PCL solution to be electrospun.

In the last two decades, electrospinning has gained a high interest as continuous nanofibers can be processed by this simple method [5,6,7,8,9,10]. The product of electrospinning is generally a fiber web in which the constituent fibers have a diameter range of 50–500 nm. Generally, electrospinning is practiced from polymer solutions, although polymer melts can also be spun. Compared to the classical fiber manufacturing techniques at electrospinning, fibers are drawn by electrostatic forces instead of mechanical ones. The formations of the fibers and the structure take place in the same time and place and therefore the technology is effective. Electrospinning technique makes possible to create composite fibers where the electrospun nanofibers play the role of the matrix. The smaller nanoparticles can be either embedded into nanofibers or they can be deposed or grafted onto their surface. In the former case, nanoparticles are admixed to the electrospinning solution. There are studies proving that carbon nanotubes [11,12,13,14,15], carbon black [16], nano-silica [17,18,19] and even metal nanoparticles [20] can be embedded.

Considering the fact that graphene sheets are larger than the diameter of usual electrospun fibers, they may be present in them in rolled, folded forms. This should be associated with a substantial reinforcing effect that is essential for our target. Alternatively, the graphene sheets may be located between the nanofibers. Nonetheless, they still fulfill the main goal, *i.e.*, acting as spacer whereby enabling good infiltration by the EP. On the other hand, an EP containing PCL nanoweb has some similarities to a system with semi-interpenetrating network structure (semi-IPN) because both phases are continuous and PCL is thermoplastic. Other criteria of semi-IPN are, however, not met; therefore this term is not used. In any case, EP with PCL nanoweb exhibits a co-continuous structure *per se*. Therefore, it is of paramount interest to check whether a thermoplastic nanoweb modified or a co-continuously structured EP has more favorable shape memory characteristics. Recall that the only difference between them, provided that the constituents are the same, is the structure of the thermoplastic phase. It is obvious that in a co-continuous morphology, achieved by phase separation during curing of the EP modified with PCL, the thermoplastic PCL should have a more fine and uniform structure than that given by the nanoweb. It is noteworthy that even semi-IPN structured systems, though not EP-based, have already been tested for shape memory performance [21].

EP-based systems with co-continuous morphology have not been tested for shape memory properties. PCL is an ideal “blending” material with EP to set co-continuous phase structure. Its application was pushed forward by the need for EP toughening. Thermoplastics of amorphous and semicrystalline nature are often used to modify EPs. Their droplets, or more sophisticated structures, covering co-continuously like those formed by phase separation upon curing of the EP, effectively trigger individual toughening mechanisms (cavitation and debonding, crack pinning, shear banding, *etc.*) [22]. Despite the *T_m_* of PCL being quite low (see later) and thus not favored for EP modification, it has been also tried as a toughening agent in EPs [23,24].

Accordingly, this work was aimed at studying the type of PCL “structuring” on the shape memory (SM) behavior of an amine cured EP. PCL was present either as nanoweb (electrospun with or without additional graphene) or as *in situ* formed constituent of a co-continuous phase structure. Information on the structures was received from scanning electron microscopy (SEM) and Raman spectrometry studies. Their SM properties were determined in tension using DMA.

## 2. Results and Discussion

### 2.1. Electrospun PCL Nanoweb

**Figure 1:**
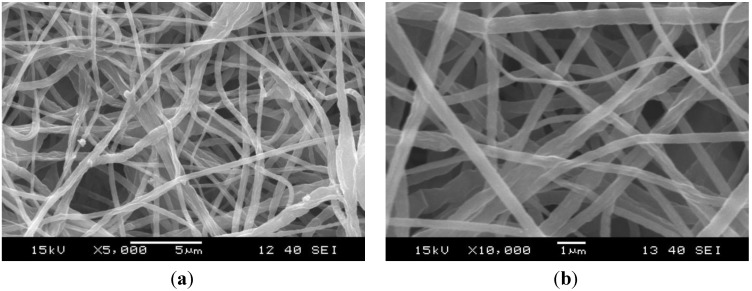
SEM pictures of polycaprolactone (PCL) nanoweb from different locations in magnification of (**a**) 5000×; and (**b**) 10,000×.

**Figure 2:**
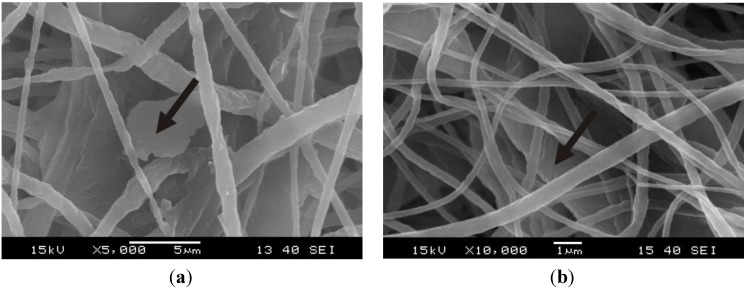
SEM pictures of PCL nanoweb/graphene from different locations in magnification of (**a**) 5000×; and (**b**) 10,000×. Note: arrows show graphene nanoplatelets.

SEM images of the morphology of the electrospun nanofibers with and without graphene can be seen in Figure 1 and Figure 2. Both PCL nanoweb with and without graphene contains fibers with diameters between 100 and 1000 nm. In case of the neat nanofibers, only a small number of beads can be observed. In the graphene containing nanofiber web, there are no beads, proving that, in this case, the viscosity of electrospinning solution was higher than that without graphene. This stabilized the liquid jet along the axis of drawing. Dispersion of graphene was effective because agglomerates cannot be found with SEM. Graphene nanoplatelets are located between nanofibers (Figure 2), but possibly also in folded form in the PCL nanofibers themselves.

### 2.2. Morphology of EP/PCL

**Figure 3:**
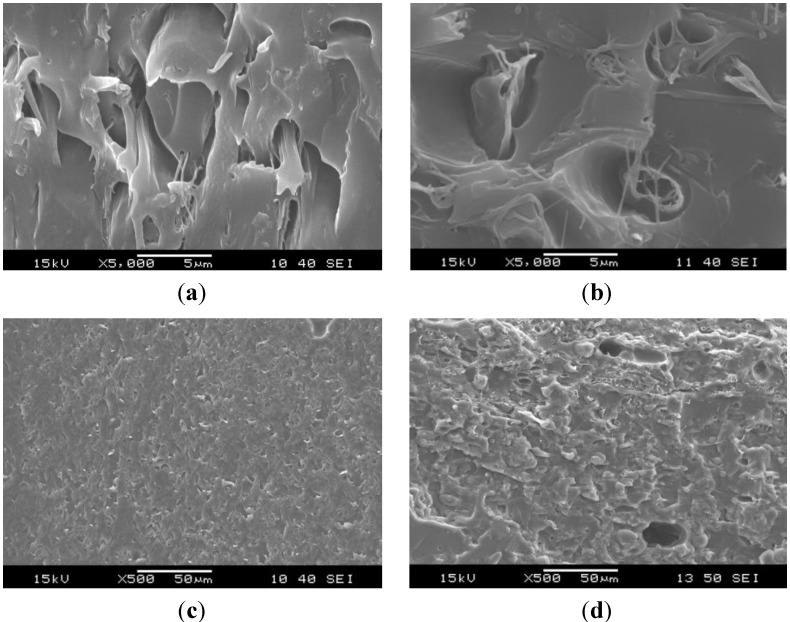
SEM micrographs of (**a,c**) cryocut; and (**b,d**) cryofractured cross sections of epoxy (EP)/PCL nanoweb.

**Figure 4:**
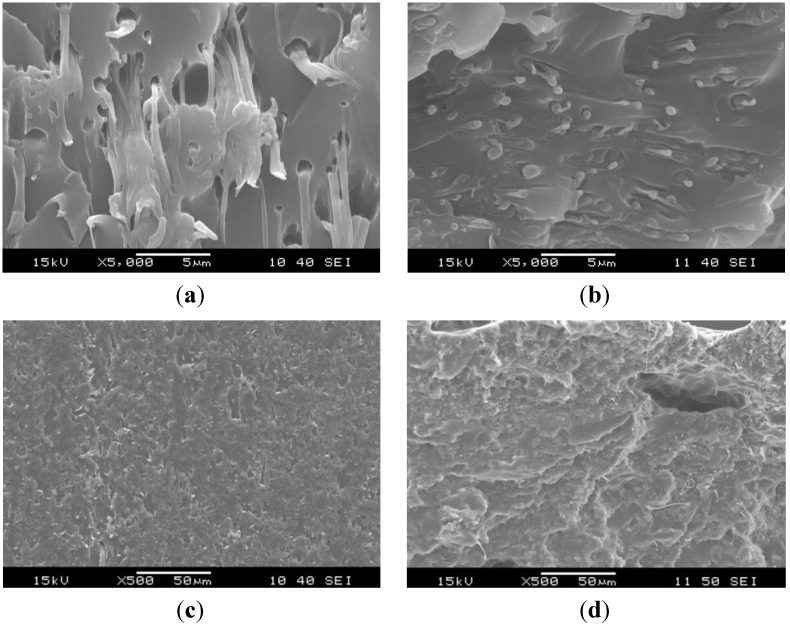
SEM micrographs of (**a,c**) cryocut; and (**b,d**) cryofractured cross sections of EP/PCL nanoweb/graphene.

The structure of the original electrospun PCL nanoweb changed after impregnation. Without graphene in PCL, the EP impregnated the PCL fibers less perfectly, which triggered their “bundling” via melting of the PCL during curing of the EP. Thus, PCL fibers formed bundles in EP/PCL nanoweb system (Figure 3). The fiber bundles have a diameter range of 1–5 μm. By contrast, hardly any “bundling” phenomenon can be resolved in the EP/PCL nanoweb with graphene (Figure 4). This confirms that graphene platelets situated between fibers acted as spacer and strengthened the nanoweb during impregnation. This supported the EP flow through the web, guaranteeing a more effective wet-out.

**Figure 5:**
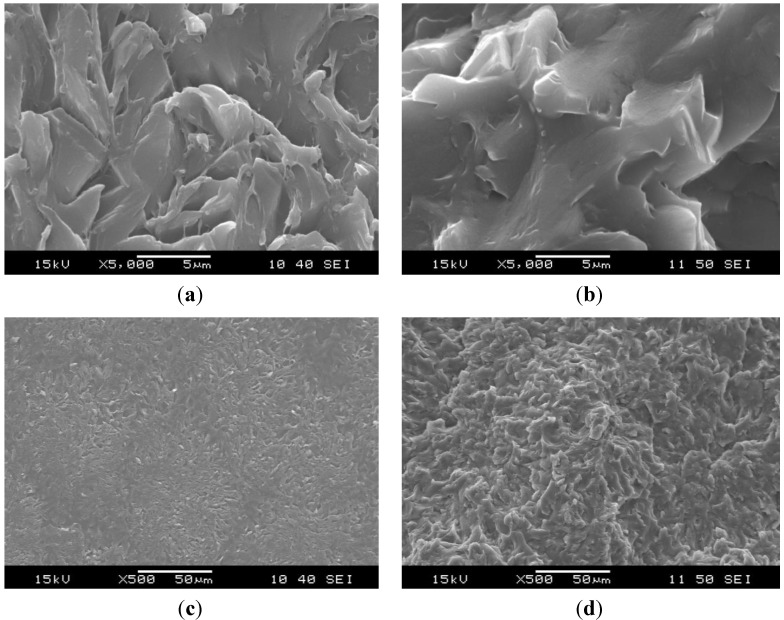
SEM micrographs of (**a,c**) cryocut; and (**b,d**) cryofractured cross sections of EP/PCL with co-continuous morphology.

**Figure 6:**
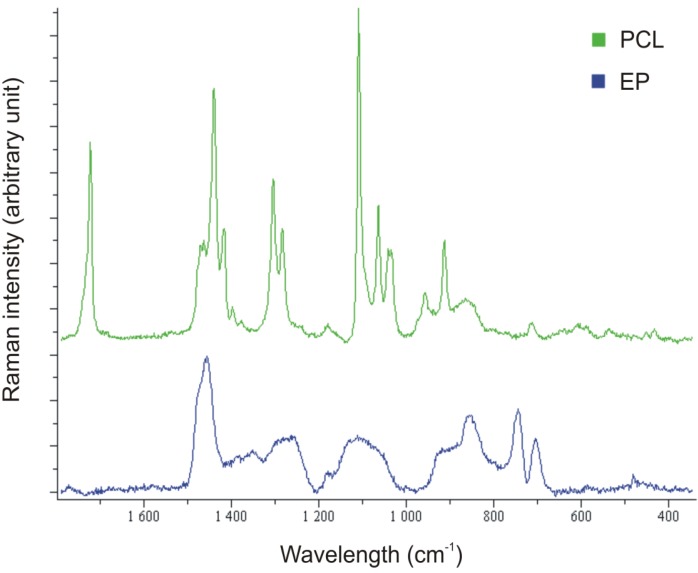
Raman spectra of EP and PCL.

**Figure 7:**
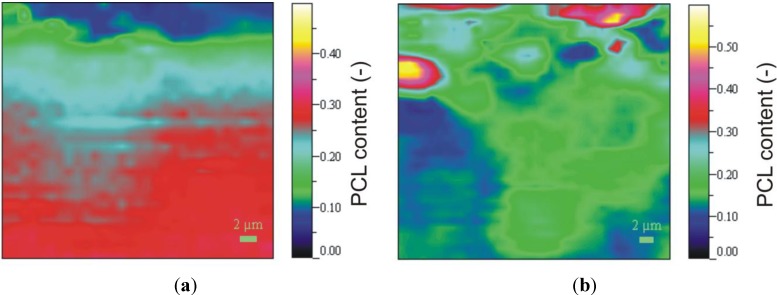
Raman maps of (**a**) EP/PCL with co-continuous morphology; and (**b**) EP/PCL nanoweb.

SEM pictures of EP/PCL with co-continuous structure (Figure 5) do not allow us to resolve the constituents. In order to estimate the characteristic dimension of the phases in this co-continuous structure, the Raman mapping technique was adapted. Figure 6 shows the reference Raman spectra of EP and PCL. Considering the reference spectra, the PCL content was “mapped” (Figure 7). Raman map of co-continuous phase structure seems to be homogeneous, meaning that the characteristic dimension of the related structure is smaller than the focus diameter of the laser beam, which was 900 nm. The size of the interpenetrating “bands” depends on the compatibility of the constituents and is usually in the range of hundreds of nanometers for thermoset IPNs [25]. In the case of nanoweb structure, the same characteristic diameter of fiber bundles can be found in the Raman map of EP/PCL nanoweb (Figure 7b) as that deduced from SEM micrographs (Figure 3).

### 2.3. Thermal and Mechanical Properties

**Figure 8:**
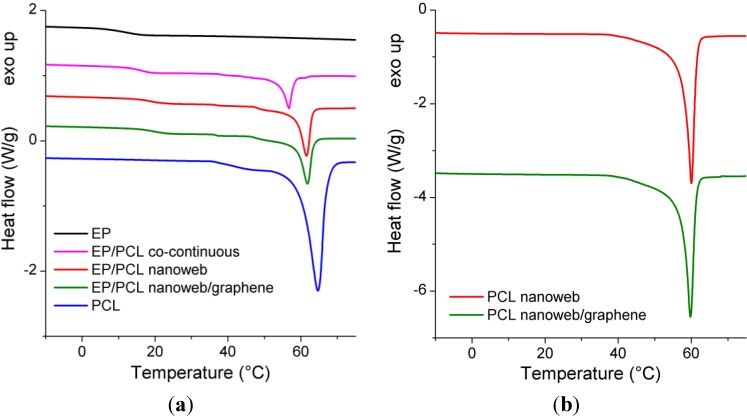
(**a**) Differential scanning calorimetry (DSC) curves of EP, PCL, EP/PCL with different structures; and (**b**) PCL nanowebs with and without graphene loading.

**Figure 9:**
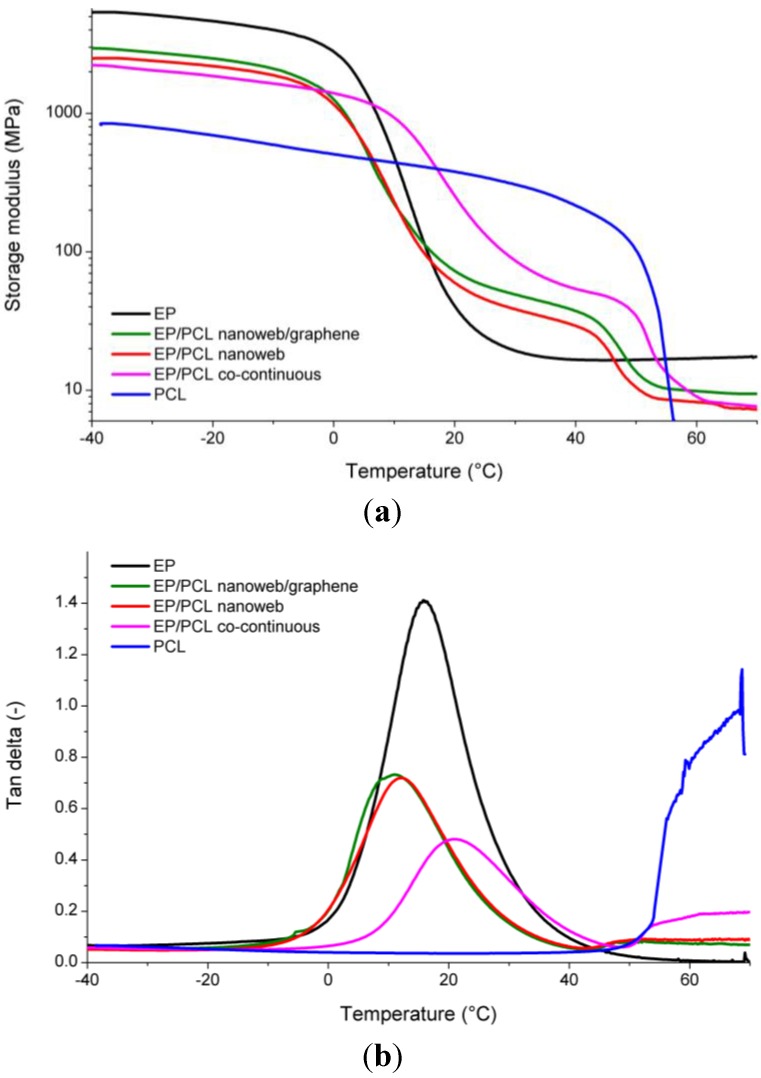
(**a**) Storage modulus; and (**b**) tanδ curves of EP, PCL and EP/PCL samples with different structures.

Phase transitions (*T_g_* of EP and *T_m_* of PCL) of the samples were determined by both DSC and DMA. DSC and DMA curves are plotted in Figure 8 and Figure 9, respectively. Numerical results of DSC and DMA are listed in Table 1. Based on these results, the deformation temperatures for SM tests were chosen. Accordingly, the first and second temporary shapes were to be set at 60 °C and 30 °C, respectively.

**Table 1:** Transition temperatures determined by DSC and DMA.

Samples	*T_g_*^DSC^ (°C)	*T_g_*^tanδ^ (°C)	*T_m_*^DSC^ (°C)	*T_m_^E′^* (°C)
EP	12	16	–	–
PCL	–	–	65	54
EP/PCL co-continuous	16	21	57	52
EP/PCL nanoweb	19	12	62	45
EP/PCL nanoweb/graphene	20	11	62	46
PCL nanoweb	–	–	56	–
PCL nanoweb/graphene	–	–	56	–

Figure 9 delivers the most persuading arguments for the presence of co-continuity for the EP/PCL systems. It is well resolved that the storage modulus of EP/PCL does not drop at the *T_g_* of the EP, which would be expected for the dispersed PCL phase. Instead, the modulus runs in between those of the PCL and EP between the *T_g_* of EP and *T_m_* of PCL (Figure 9a). No clear plateau appears in this temperature range, reflecting the thermoplastic character of the PCL. Nevertheless, the load bearing capacity is given by the continuous PCL phase by whatever means achieved (nanoweb,* in situ* curing). The fact that the tanδ values of the EP/PCL systems are between the parent components above *T_m_* of PCL also supports the co-continuity (Figure 9b). A further peculiar feature is that incorporation of nanoweb (with and without graphene) shifted the *T_g_* of EP toward slightly lower temperatures, whereas an adverse tendency was recognizable for the phase separated in *in situ* cured EP/PCL (Figure 9b). This may be due to a combination of chemical (reactive end groups of PCL) and mechanical constraint effects which were, however, not investigated.

**Figure 10:**
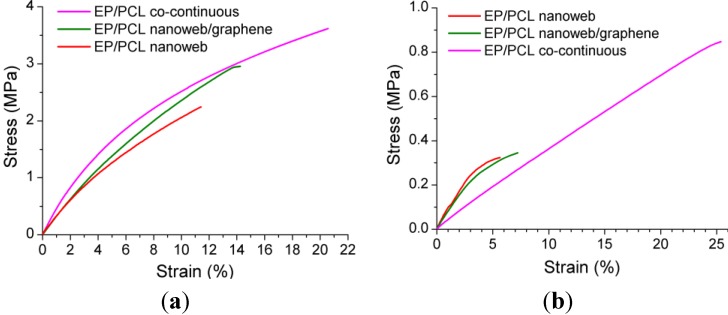
Stress-strain curves of EP/PCL samples with different structures at (**a**) 30 °C; and at (**b**) 60 °C.

Deformability of the samples at the selected deformation temperatures were measured in tensile mode in the DMA device. Stress-strain curves are depicted in Figure 10. EP/PCL with co-continuous morphology has the highest strain at break in case of both temperatures. This is due to the better homogeneity and lower void content of the sample. Impregnation is always a critical step in preparation of composites when a preformed structure should be wet out. Recall that EP/PCL with co-continuous morphology was produced* in situ* via phase separation upon curing. For the first temporary shape (ε*_m_*_1_) of the SM test, 2% strain was selected, because below this strain all the stress-strain curves, measured at 60 °C, were linear (Figure 10b). For the second temporary shape (ε*_m_*_2_) a strain was chosen that was two times higher than ε*_m_*_1_.

### 2.4. Triple-Shape Memory Properties

**Figure 11:**
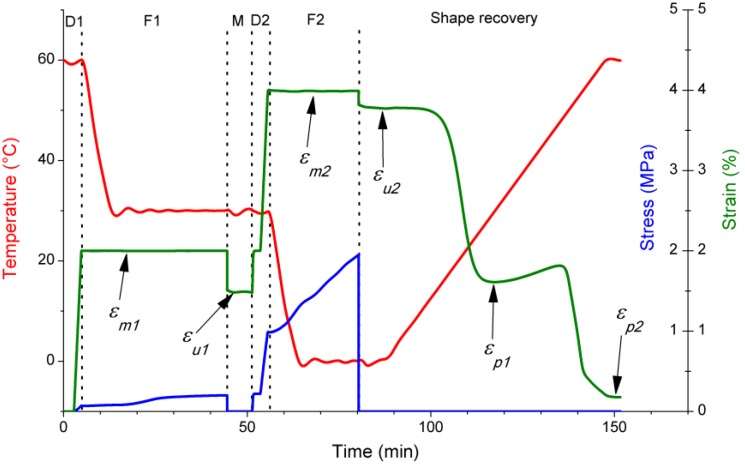
Temperature, stress and strain curves of EP/PCL nanoweb sample during SM test. Arrows indicate the strain values used for calculations. Dotted lines separate the steps of the test; where D1, F1, M, D2, F2 mean first deformation; first fixation; measurement of the first fixed shape; second deformation; and second fixation, respectively.

Triple-shape memory tests were performed in Q800 DMA device. Registered temperature, stress and strain values in function of time are presented for the sample EP/PCL nanoweb in Figure 11.

SM properties corresponding to EP phase [shape fixity ratio of the second temporary shape (*R_f_*_2_) and shape recovery ratio of the first fixed temporary shape (*R_r_*_1_)] are quite similar for all samples. Note, that all the samples contained *ca.* 23 wt % PCL. The SM properties depending on PCL phase [shape fixity ratio of the first temporary shape (*R_f_*_1_) and shape recovery ratio of the original shape (*R_r_*_1_)] are, however, quite different for the studied systems. These SM properties for EP/PCL nanoweb with graphene are worse than those without. EP/PCL with co-continuous structure can fix the first temporary shape better than the EP/PCL nanoweb. On the other hand, the latter one can recover the original shape slightly better than the co-continuously structured EP/PCL. Calculated SM properties can be found in Table 2. Value ranges are given instead of average results, when the repeated test did not give the same result as the first one.

**Table 2:** Results of the triple-shape memory tests. Tests were conducted on two parallel specimens.

Samples	*R_f_*_1_ (%)	*R_f_*_2_ (%)	*R_r_*_1_ (%)	*R_r_*_2_ (%)
EP/PCL nanoweb	67–74	94–95	94–96	89
EP/PCL nanoweb/graphene	61–67	95	95	69–94
EP/PCL co-continuous structure	81–82	94–95	94	85

## 3. Experimental Section

### 3.1. Materials

PCL granule (grade CAPA 6800) was supplied by Solvay (Brussels, Belgium) having a molecular weight of 80 kDa and *T_m_* of 58–60 °C. Dichloromethane and ethanol solvents were purchased from Molar Chemicals (Budapest, Hungary). The graphene nanoplatelets (grade xGnP) used were from XG Sciences (Lansing, MI, USA). Ipox MR3012 type EP resin (Ipox Chemicals, Budapest, Hungary) and Jeffamine D230 type amine hardener (Huntsman, Bergkamen, Germany) were chosen, because their stoichiometric mixture provides a cured EP with a *T_g_* of around ambient temperature [26]. Note that this *T_g_* is considerably lower than the *T_m_* of PCL which was essential to achieve the required triple-shape memory.

### 3.2. Electrospinning

PCL granule was dissolved in a mixture of dichloromethane/ethanol in a mass ratio of 7:3. The solution was stirred by magnetic mixer for 6 hours at ambient temperature for complete dissolution. The PCL concentration was 3.5 wt %. Another solution with additional graphene of 3.5 wt % relative to the PCL content was also produced. In this case the graphene was dispersed as follows. The graphene was admixed to the solvent mixture, and then it was treated for 20 min by Bandelin Sonoplus HD2200 type (Bandelin, Berlin, Germany) ultrasonic homogenizer equipped with an UW 2200 type ultrasonic converter of the same company. After the treatment, the PCL was mixed to the solution and the ultrasonic treatment was repeated. The adequate dispersion of the graphene was empirically confirmed by the increase of the solution viscosity.

Electrospinning was carried out by the classical, single capillary setup [16] in a bottom to top vertical arrangement. The applied capillary spinneret was a hypodermic needle that was connected to a DC high voltage power supply. The solution was fed through the capillary by a SEP-10S Plus (Aitecs, Vilnius, Lithuania) type syringe pump. The applied voltage, the distance between the grounded metal plate electrode and the spinneret and the constant solution feed rate was set to 40 kV, 100 mm and 16 mL/h, respectively in both cases. Layers of PCL nanowebs, having a thickness of 0.1–0.4 mm, were produced from both neat and graphene-containing solutions.

### 3.3. Specimen Preparation

The ratio of EP resin and hardener was stoichiometric. For their mixing a radial stirrer was used. In order to remove the residual air bubbles, the resin was put in a desiccator and vacuum was applied.

Preparation of PCL nanoweb-containing EP samples took place in an open mold made of polytetrafluoroethylene (PTFE). Two layers of nanofiber mats were placed into the mold and then hermetically sealed by polyamide foil prior to the impregnation via vacuum assisted resin transfer molding. As the PCL has low *T_m_*, the EP was cured at ambient temperature for 48 hours. Afterward, the EP/PCL nanoweb composites were removed from the mold and post-cured at 80 °C for 2 hours. At this temperature the nanofibers were molten and after their recrystallization the web obtained its final structure. The composite samples had a thickness range of 0.2–1.0 mm, from which 25 mm long and 7 mm wide specimens were cut.

EP/PCL sample with co-continuous morphology, containing 23 wt % PCL, was made as follows. PCL granule was dissolved in the EP resin by mixing at 100 °C for one hour, and then cooled to 70 °C. Afterward, hardener was added, and mixed for 2 min. This mixture was cast into the PTFE mold, the entrapped air removed in vacuum desiccator (“aerated” 3 times), and cured at ambient temperature for 48 hours followed by post curing at 80 °C for 2 h.

### 3.4. Morphology Determination Techniques

The morphology of the samples was studied by scanning electron microscopy (SEM) using a JEOL JSM-6380LA device (Jeol, Tokyo, Japan). Specimens were cryomicrotomed with a glass knife at −80 °C using a Leica EM UC6 microtome (Leica Microsystems, Wetzlar, Germany) equipped with a cryochamber. Specimens were cryofractured, as well. The cryocut and cryofractured surfaces were sputtered with Au/Pd alloy before SEM inspection.

The cryofractured surfaces were also analyzed by Raman spectrometry. The Raman spectra have been recorded using a Horiba Jobin-Yvon (Lyon, France) LabRam system with a 532 nm Nd: YAG laser (Sacher Lasertechnik, Marburg, Germany) and an Olympus BX-40 optical microscope (Olympus, Hamburg, Germany). An objective of 50× magnification was used for spectrum acquisition. In this case the diameter of laser beam in focus point is 900 nm. Spectrum acquisition time was 20–30 s and two spectra were averaged at each pixel. The step size between adjacent pixels was 1 μm along both axes. Details of the used Raman mapping technique are available elsewhere [27].

### 3.5. Thermal and Mechanical Analysis

Differential scanning calorimetry (DSC) was carried out in a Q2000 type DSC device (TA Instruments, New Castle, DE, USA) using aluminum sample holders. Heating rate was set to 10 °C/min, temperature range was between −30 °C and 80 °C. From DSC curve (heatflux as a function of temperature) *T_g_* of EP and *T_m_* of PCL were determined as the median of baseline change and peak temperature of melting, respectively. DSC curves shown represent the first heating.

DMA and tensile test were conducted in a DMA Q800 device (TA Instruments) in tension mode, where clamping distance was 12 mm and clamping force was similar for each specimen, verified by a torque screw. In the case of DMA heating rate, oscillation frequency and oscillation amplitude were 3 °C/min, 10 Hz and 0.05%, respectively. From DMA curves (storage modulus or tanδ as a function of temperature) *T_g_* of EP and *T_m_* of PCL were read as peak temperature of tanδ and onset temperature of storage modulus’ drop, respectively. Tensile tests were performed at both 30 and 60 °C. Force rate of tension was set to 3 N/min. DSC, DMA and tensile tests were conducted on single specimens.

### 3.6. Triple-Shape Memory Behavior

In order to test SM properties also the Q800 DMA device was used in tension mode. Clamping distance was 12 mm and clamping force was similar for each specimen. During SM tests the specimens were deformed at 60 °C to ε*_m_*_1_ = 2% strain and immediately cooled to 30 °C, while ε*_m_*_1_ was maintained. After 30 min, the stress was reduced to zero and the first fixed temporary shape (ε*_u_*_1_) was measured. Specimens were deformed at 30 °C further to ε*_m_*_2_ = 4% strain and immediately cooled to 0 °C, while ε*_m_*_2_ was maintained. After 15 min, the stress was reduced to zero and the second fixed temporary shape (ε*_u_*_2_) was recorded. After this programing procedure specimens were heated up to 60 °C with a heating rate of 1 °C/min. During reheating, the stress was set to zero and the strain was measured. The latter formed a double sigmoid in function of time having a plateau, which is the recovered first temporary shape (ε*_p_*_1_) around 30 °C and ending in the recovered original shape (ε*_p_*_2_) around 60 °C.

**Figure 12:**
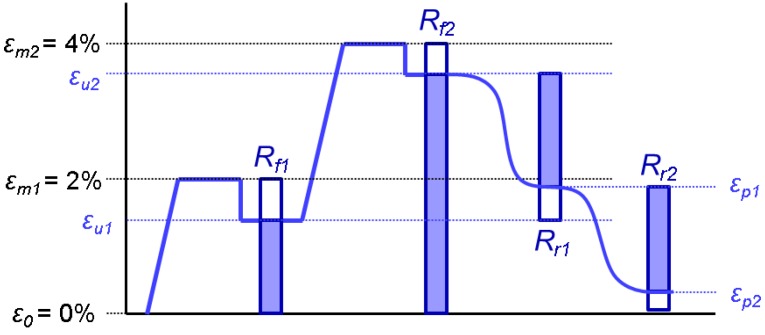
Schematic diagram of triple-shape memory test, where horizontal axis is time and vertical axis is strain.

Abilities to fix the first and second temporary shapes, and to recover the first fixed shape and the original shape, were characterized with shape fixity ratios *R_f_*_1_ and *R_f_*_2_, and shape recovery ratios *R_r_*_1_ and *R_r_*_2_, respectively. These ratios are calculated according to Equations (1–4), in which meaning of symbols are listed in Table 3. Figure 12 demonstrates graphically the triple-shape memory cycle and the mentioned strain values and ratios.

(1) $$R_{f1}=\frac{\varepsilon_{u1}-\varepsilon_{0}}{\varepsilon_{m1}-\varepsilon_{0}}$$

(2) $$R_{f2}=\frac{\varepsilon_{u2}-\varepsilon_{0}}{\varepsilon_{m2}-\varepsilon_{0}}$$

(3) $$R_{r1}=\frac{\varepsilon_{u2}-\varepsilon_{p1}}{\varepsilon_{u2}-\varepsilon_{u1}}$$

(4) $$R_{r2}=\frac{\varepsilon_{p1}-\varepsilon_{p2}}{\varepsilon_{p1}-\varepsilon_{0}}$$

**Table 3:** Meaning of symbols appearing in Equations (1–4).

Symbol (unit)	Meaning
*R_f_*_1_ (%)	Shape fixity ratio of fixing the first temporary shape.
*R_f_*_2_ (%)	Shape fixity ratio of fixing the second temporary shape.
*R_r_*_1_ (%)	Shape recovery ratio of recovering the first fixed shape.
*R_r_*_2_ (%)	Shape recovery ratio of recovering the original shape.
ε_0_ (%)	Original or initial shape, which is zero in our case.
ε*_m_*_1_ (%)	Required first temporary shape, which is 2% in our case.
ε*_m_*_2_ (%)	Required second temporary shape, which is 4% in our case.
ε*_u_*_1_ (%)	Fixed first temporary shape, which was measured.
ε*_u_*_2_ (%)	Fixed second temporary shape, which was measured.
ε*_p_*_1_ (%)	Recovered first temporary shape, which was measured.
ε*_p_*_2_ (%)	Recovered original shape, which was measured.

## 4. Conclusions

This work was devoted to produce triple-shape memory epoxy (EP)/polycaprolactone (PCL) systems with different phase structures at the same PCL content (23 wt %) and to study the effect of their structure on the triple-shape memory behavior. The three structurally different systems, analyzed by scanning electron microscopy and Raman spectrometry, were:

1. EP/PCL nanoweb: EP could not fully penetrate in between the web forming fibers due to processing-induced “compaction”. Therefore, the fibers tended to form bundles with diameters of 1–5 μm after post curing conducted above the melting temperature of PCL;
2. EP/PCL nanoweb with graphene: graphene nanoplatelets, located also between the fibers, likely acted as spacers and strengthened the nanoweb structure during impregnation. As a consequence, the infiltrating EP could wet out the fibers well, and no cure temperature-induced “bundling” phenomenon was observed;
3. EP/PCL with co-continuous structure: both phases are continuous and the characteristic dimension of the “intermingling bands” is most likely below 900 nm.

Based on the results achieved with specimens subjected to triple-shape memory test in tension mode, the following conclusions can be drawn:

1. Shape memory properties, related to the EP phase, are similar for all samples, irrespective of their structure;
2. Shape memory properties belonging to PCL phase are worsened by incorporation of grapheme;
3. EP/PCL with co-continuous morphology possessed the best triple-shape memory properties. Therefore, attention should be focused on co-continuously structured EP-based systems for achieving triple-shape memory performance.
